# Modulating the Ubiquitin–Proteasome System: A Therapeutic Strategy for Autoimmune Diseases

**DOI:** 10.3390/cells11071093

**Published:** 2022-03-24

**Authors:** Dhananjay Yadav, Ji Yeon Lee, Nidhi Puranik, Pallavi S. Chauhan, Vishal Chavda, Jun-O. Jin, Peter C. W. Lee

**Affiliations:** 1 Department of Medical Biotechnology, Yeungnam University, Gyeongsan 38541, Korea; dhanyadav16481@gmail.com; 2 Research Institute of Cell Culture, Yeungnam University, Gyeongsan 38541, Korea; 3 Division of Rheumatology, Department of Medicine, Seoul St. Mary’s Hospital, Catholic University, Seoul 06591, Korea; jylee217@catholic.ac.kr; 4 Department of Biochemistry & Genetics, Barkatullah University, Bhopal 462026, Madhya Pradesh, India; nidhipuranik30@gmail.com; 5 Department of Life Sciences, ITM University, Gwalior 474001, Madhya Pradesh, India; pallavi.chauhan97@gmail.com; 6 Department of Pathology, Stanford School of Medicine, Stanford University Medical Center, Stanford, CA 94305, USA; chavdavishal2@gmail.com; 7 Department of Biomedical Sciences, University of Ulsan College of Medicine, Asan Medical Center, Seoul 05505, Korea; 8 Lung Cancer Research Center, University of Ulsan College of Medicine, Asan Medical Center, Seoul 05505, Korea

**Keywords:** autoimmune disease, central nervous system, multiple sclerosis, UPS, therapeutic target, 26S proteasome, E3 ligase

## Abstract

Multiple sclerosis (MS) is an autoimmune, neurodegenerative disease associated with the central nervous system (CNS). Autoimmunity is caused by an abnormal immune response to self-antigens, which results in chronic inflammation and tissue death. Ubiquitination is a post-translational modification in which ubiquitin molecules are attached to proteins by ubiquitinating enzymes, and then the modified proteins are degraded by the proteasome system. In addition to regulating proteasomal degradation of proteins, ubiquitination also regulates other cellular functions that are independent of proteasomal degradation. It plays a vital role in intracellular protein turnover and immune signaling and responses. The ubiquitin–proteasome system (UPS) is primarily responsible for the nonlysosomal proteolysis of intracellular proteins. The 26S proteasome is a multicatalytic adenosine-triphosphate-dependent protease that recognizes ubiquitin covalently attached to particular proteins and targets them for degradation. Damaged, oxidized, or misfolded proteins, as well as regulatory proteins that govern many essential cellular functions, are removed by this degradation pathway. When this system is affected, cellular homeostasis is altered, resulting in the induction of a range of diseases. This review discusses the biochemistry and molecular biology of the UPS, including its role in the development of MS and proteinopathies. Potential therapies and targets involving the UPS are also addressed.

## 1. Introduction

Paul Ehrlich coined the term “horror autotoxicus” at the turn of the 20th century to describe the processes through which the immune system assaults the body. Five decades later, Burnet et al. identified autoantibodies, thus providing a theoretical basis for auto-reactivity [1]. This concept is currently recognized as a flaw in the immune system involving B or T cells and is implicated in adaptive immunological activation, which can lead to autoimmune disorders causing tissue damage.

There are two types of tolerance of the immune system for self-antigens: central and peripheral. Central tolerance is induced in the thymus, with medullary thymic epithelial cells and medullary dendritic cells presenting a wide range of self-peptides on their surfaces along with specialized proteins that growing T cells recognize as major histocompatibility complexes (MHC). T cells with T cell receptors that recognize a self-peptide in the milieu of an MHC molecule, with an affinity greater than a particular threshold, are excluded by negative selection, which prevents autoreactive T lymphocytes from causing injury to host tissues [2]. Ubiquitination has been linked to the development and maintenance of self-tolerance, as well as the control of autoreactive immune cell proliferation [3].

## 2. Multiple Sclerosis (MS)

**Figure 1:**
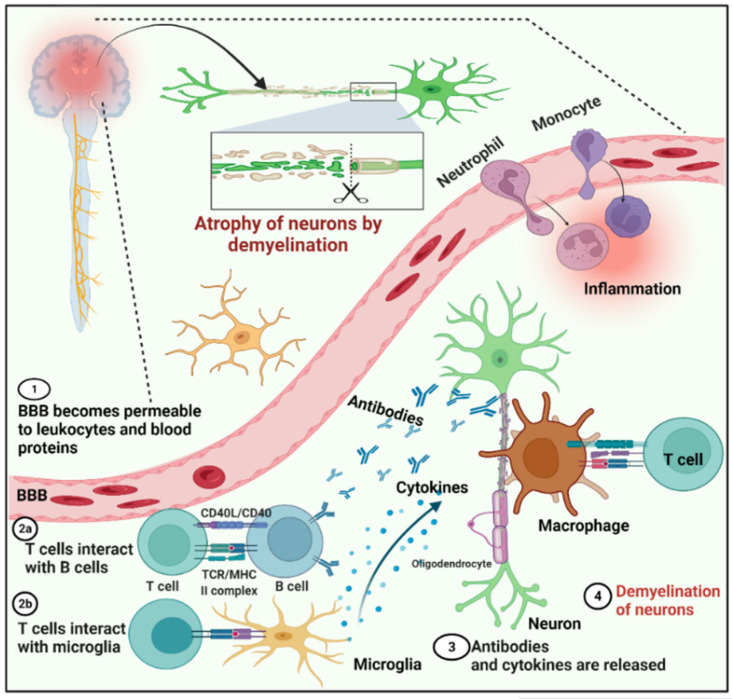
General schematic representation of the mechanism of the autoimmune disorder multiple sclerosis. Steps: 1, The blood–brain barrier becomes permeable to leukocytes and blood proteins. 2, T cells interact with B cells, and T cells simultaneously interact with microglia and induce both types of cells. 3, In response to T cells, microglia and B cells produce cytokines and antibodies, respectively, against myelin. 4, Neuron demyelination occurs.

Multiple sclerosis is an autoimmune, demyelinating inflammatory disorder of the central nervous system (CNS) that has various clinical and pathological manifestations [4,5,6]. Autoantibodies function in the immunopathogenesis of MS; for example, autoantibodies are intrathecally synthesized after clonal proliferation [4]. Multiple sclerosis causes visual abnormalities, paresthesia (unusual skin sensations), ataxia, and muscle weakness due to CNS inflammation and is the primary cause of disability in young adults in Western countries [7]. Perivenular inflammatory lesions that develop into demyelinating plaques characterize MS. A small proportion of T lymphocytes, especially the MHC class I-restricted CD8+ type, are found in inflammatory infiltrates, such as B cells and plasma cells (Figure 1). Damage to oligodendrocytes and demyelination is caused by inflammation. The axons are mostly intact during the early stages of the disease, but irreparable axonal damage develops as the disease advances [8]. One of the most significant structural components of the myelin sheath, which protects axons and accelerates nerve impulse transmission, is myelin basic protein (MBP). This protein was discovered as a major autoantigen in MS, in addition to myelin oligodendrocyte glycoprotein. MBP and its peptides have been investigated for decades as key players in the autoimmune response and as encephalitogenic agents [3]. Figure 1 shows the mechanism of MS.

## 3. Proteinopathies

Proteinopathies are a set of neurological disorders characterized by protein mutations, aggregation, and misfolding in brain cells. The UPS, which regulates protein turnover, has a critical role in the pathogenesis of neurological diseases [9]. Misfolded proteins that collect within the brain due to disease-related gene mutations or faulty protein homeostasis are frequently associated with neuronal death. The UPS and the autophagy-lysosomal route are two key degradation processes that remove undesirable or misfolded proteins from cells, thus preventing their cellular accumulation and maintaining cell viability. Both of these degradative processes rely on the ubiquitin modification of targets [10,11].

The accumulation of ubiquitin, proteasomes, and ubiquitin conjugates is associated with specific disease-defining proteins and can result in various chronic neurodegenerative diseases [12]. However, a direct pathogenic connection with anomalies in the ubiquitin system has not been firmly established for any of these diseases. A complicating factor is the finding that most of these illnesses, including Alzheimer disease (AD) and Parkinson disease (PD), are syndromes with completely different etiologies [13]. The accumulation of ubiquitin conjugates in Lewy inclusion bodies might be secondary in these diseases, as many attempts based on the UPS have been unable to remove the abnormal proteins [14]. If the first hypothesis is that inclusion bodies form due to the tendency of abnormal proteins to aggregate, then the process involves an active cellular mechanism [15].

The pathogenesis of protein aggregation remains unclear. The UPS can be inhibited by aggregated proteins. However, a new concept has been introduced, which states that the aggregation of cytosolic and nucleoplasmic proteins isolates them from the cellular machinery and is therefore protective [16]. Although the mechanism involved in the formation of inclusion bodies containing disease-specific aggregated proteins is common to several neurodegenerative diseases, it is poorly understood [17].

## 4. The UPS

A group of scientists won the Nobel Prize in Chemistry for their elucidation of the function of ubiquitin in 2004. Ubiquitin is a component of the UPS, which destroys >80% of all intracellular proteins, both normal and pathological. The 26S proteasome is a dynamic multi-subunit proteolytic complex with a molecular weight of 2.5 MDa [18]; it is the principal enzyme in eukaryotes for non-lysosomal-based protein degradation. This allows for the maintenance of proteostasis, namely, the balance and optimal biological function of cellular proteins. Denatured, crushed, misfolded, and unwanted protein moieties are removed by proteasomal degradation, which also helps regulate the amounts of essential proteins responsible for cell growth, such as cyclins and transcription factors [19]. The proteasome holoenzyme comprises a 20S core particle and one or two 19S regulatory particles [20].

The UPS starts with three enzymes working together to attach a tiny protein called ubiquitin to a protein substrate to target it for destruction. A cascade of three ubiquitin-modifying enzymes catalyzes ubiquitination: ubiquitin-activating enzymes E1s, ubiquitin-conjugating enzymes E2s, and ubiquitin-ligases E3s. In the first step, an E1 activates ubiquitin in the presence of ATP, establishing a thioester link between ubiquitin’s C-terminal glycine and E1’s cysteine sulfhydryl group. The activated ubiquitin is then transported to a cysteine residue in an E2’s active site, which defines the kind of substrate for ubiquitination. Ubiquitin is covalently linked to the substrate in the last stage, which is coordinated by a particular E3 that dictates the specificity of the substrate. The three-step process allows the target substrates to be ubiquitinated quickly and efficiently, allowing cells to adjust to changes in minutes [7].

**Figure 2:**
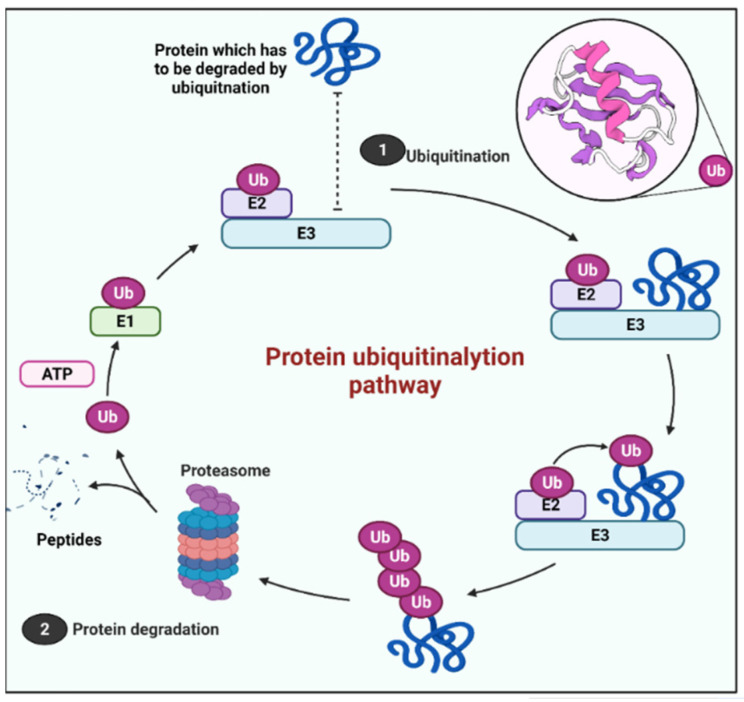
Schematic representation of the protein degradation process by the ubiquitin proteasome system.

Thereafter, the system depends on a large multicatalytic proteolytic molecule called the 26S proteasome, which degrades the ubiquitin (Ub)-tagged protein. The 26S proteasome is an enzyme with various catalytic sites, including a 20S core proteasome and regulatory 19S complexes. After recognizing a tagged protein, proteasome-associated deubiquitinases (DUBs) recycle the ubiquitin chains, then the substrate is moved to the inner proteolytic cavity and digested into short peptides that can be presented on the cell surfaces of related cells for immunosurveillance, or they can be chopped into free amino acids [21] (Figure 2).

Ubiquitination is also a reversible post-translational modification that plays a crucial role in signal transmission and protein stability. DUBs are the main enzymes involved in the reversal process. Over 100 DUBs in humans have been classified into the following families: ubiquitin C-terminal hydrolases, ubiquitin-specific proteases (USPs), ovarian tumor proteases (OTUs), Josephins, and JAB1/MPN/MOV34 metalloenzymes (also known as MPN). To discriminate among the various ubiquitin-like entities, DUB activity is extremely selective and regulated at many levels.

The UPS is associated with the pathogenesis of PD, AD, rheumatoid arthritis (RA), amyotrophic lateral sclerosis (ALS), Huntington disease, prion diseases, and other neurological disorders. The 26S proteasome is a promising target for the treatment of autoimmune disorders. Circulating proteasomes and antiproteasome autoantibodies have been identified in blood samples from patients with autoimmune diseases, such as systemic lupus erythematosus (SLE), MS, and RA. Proteasome inhibitors (PIs) such as carfilzomib and bortezomib are approved anticancer drugs that selectively inhibit 26S proteasomes. Both compounds decrease autoantibodies and halt illness development in animal models of SLE [22,23].

## 5. Therapeutic Targets in UPS

The UPS plays a central role in basic cellular processes, which causes difficulties in the development of drugs that modulate its activity [24]. Inhibiting enzymes that are common throughout the pathway affect many beneficial and toxic processes. PIs can be useful for some diseases [25]. In fact, a specific PI has been approved for use against multiple myeloma (a malignant tumor of immune plasma cells). Drugs can act against neoplasms by inhibiting cell cycle inhibitors or various antiapoptotic transcriptional regulators and also act as neuroprotectants by inhibiting NF-κB activation [26]. Self-peptide presentation is inhibited in autoimmune diseases, together with disrupted signal transmission along cellular immune cascades [27]. Belogurov et al. (2014) discovered that the 26S proteasome degrades the myelin multilayered membrane sheath and MBP in mammalian cells in a ubiquitin-independent manner [3].

### 5.1. Targeting E3 in Proteinopathies

The final step of ubiquitin conjugation is catalyzed by E3s, which transfer ubiquitin from ubiquitin-conjugating enzymes (E2s) to substrates [28]. There are an estimated 600–700 E3 ligase genes representing ~5 percent of the human genome. Mutations in E3 ligase genes have been found in a variety of neurological disorders, which is not surprising [29]. Recent advances in drug development include the binding of small molecules to specific E3s, ultimately leading to their inhibition [30]. Several substrates with phosphorylation target sites spanning E3 inhibition sites are of interest [31]. When phosphorylation destabilizes negative regulators such as p27 and IkBα, E3 inhibition can control irregular cell turnover and reduce unwanted activity of the immune system [32].

Ubiquitination plays an essential role in determining the fate of proteins, as well as the post-translational regulation of gene expression. E3 ubiquitin ligases are crucially involved in ubiquitination [33]. These ligases attach a Ub moiety to target proteins, tagging them for proteasome-dependent destruction. Various types of E3 Ub ligases are target-protein-specific (Table 1) [34].

Helical filament binding to tau (PHF-tau) is altered by polyubiquitins in AD [43]. The lysine residue of PHF-tau accepts ubiquitin at CHIP, an E3 ubiquitin ligase of PHF-tau [44]. Lysine residues of APP conjugated with ubiquitin in the mouse brain leads to Aβ40 accumulation [45].

**Figure 3:**
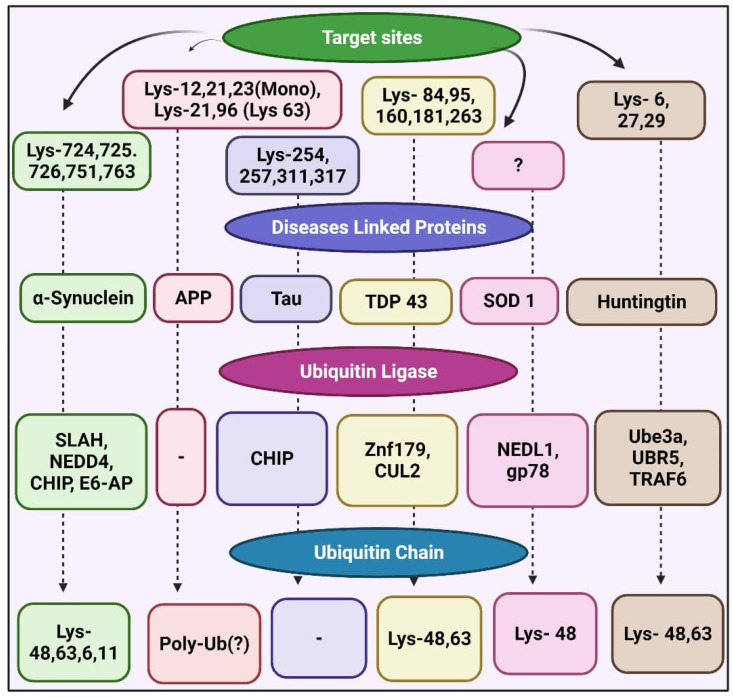
Ubiquitination of neurodegenerative-disease-associated proteins and various target sites [48].

In PD, E3 ubiquitin ligases ubiquitinate α-synuclein. Seven in absentia homolog, an E3 ubiquitin ligase, leads to α-synuclein monoubiquitination at specific Lys residues, which increases α-synuclein aggregation and apoptosis [46]. The ubiquitin ligase NEDD4 also targets α-synuclein, resulting in Lys-63-polyubiquitin [47]. The endolysosomal pathway leads to the degradation of ubiquitin-α-synuclein, suggesting that it might protect against PD pathogenesis. Like PHF-tau, mono- or polyubiquitination involves CHIP, whereas USP9X leads to the deubiquitination of CHIP-monoubiquitinated α-synuclein [48]. The knockdown of USP9X prompts the accumulation of monoubiquitinated α-synuclein, and proteolytic inhibition intensifies the formation of α-synuclein inclusions [10]. Lewy bodies in the brain of PD patients contain the ubiquitin ligase E6-AP and lead to α-synuclein ubiquitination followed by proteasome-dependent degradation (Figure 3) [49].

Antiubiquitin antibodies detect Znf179-ubiquitin-ligase-targeted TDP-43 and SOD1, which cause ALS [50], in three major regions of the brain, namely the cortex, hippocampus, and midbrain. The knockout of Znf179 suppresses the proteasomal increase of TDP-43, which leads to insoluble TDP-43 accumulation and the cytosolic inclusion of TDP-43 [51]. Ubiquitin ligase CUL2 can modify the misfolded polyubiquitin TDP-43, which is coordinated with the von Hippel Lindau protein [52].

The ubiquitination sites Lys-84, -95, -160, -181, and -263 in TDP-43 have been identified by mass spectrometry [53]. The ubiquitin ligases NEDL1 and gp78 target SOD1 and NEDL1, and SOD1 inclusions colocalize in motor neurons of the ventral horn of the spinal cord of patients with ALS and in transgenic SOD1 mutant mice. The ubiquitin ligase gp78 also plays a role in SOD1 ubiquitination [54].

The Gp78 protein comprises at least five transmembrane domains, including a consensus finger RING sequence; it plays a major role in endoplasmic-reticulum-associated protein degradation and causes ataxin-3 ubiquitination [55,56]. The overexpression of Gp78 promotes SOD1 and ataxin-3 ubiquitination in cultured cells, whereas eliminating gp78 stabilizes them [57].

Lys-48- and 63-polyubiquitin regulates huntingtin turnover. Clusters of the huntingtin mutant include Lys-63 polyubiquitin chains [58]. The Huntington’s disease (HD) protein is ubiquitinated differently by K48 and K63 in ubiquitin. Huntingtin breakdown is promoted by K48-mediated ubiquitination, whereas huntingtin aggregation is accelerated by K63-mediated ubiquitination. The ubiquitination mediated by K-48 is dependent on Ube3a, whose expression decreases with age. Bhat et al., in a study on a mouse model, observed that in the elderly HD KI mouse brain, overexpression of Ube3a can diminish mutant huntingtin accumulation and aggregation [59]. The UBR5 ubiquitin ligase mediates the Lys-48 proteasomal degradation of normal and mutant huntingtin [60].

The huntingtin mutant containing the Lys-63-ubiquitin chain can be stimulated by tumor-necrosis-factor-receptor-associated factor 6, which contributes to its autophagic clearance [61]. Ubiquitin ligase causes the ubiquitination of several neurodegenerative-disease-associated proteins and removes pathogenic proteins (Figure 3) [62].

E3 ligases control disease pathogenesis in various ways by regulating the levels of susceptible proteins. Hence, defining new targets of E3 ligases and identifying new E3 ligases will provide detailed molecular information that will deepen our understanding of the pathogenesis of several neurological disorders and lead to the development of new therapies [63].

### 5.2. Potential Therapeutic Targets in MS

Multiple sclerosis is a CNS-associated disease that causes visible muscle dysfunction and impairment. Its major impact is on the brain and spinal nerves [64]. Research studies on the pathophysiology of MS showed that the UPS plays a significant role [65,66]. The localization of Ub conjugates in patients with MS might indicate UPS deterioration. Although the UPS participates in the pathogenesis of MS, details of the relationship between UPS and MS remain unknown [67]. Increased functional repression of the UPS and of interferon beta-1b (IFN-β1b) levels results in the development of MS. A better understanding of the molecular network of the UPS should provide more insight into the pathogenesis of MS. Myeloid leukemia 1 (MCL-1) is regulated by the UPS and is upregulated in MS [68].

Type-I interferon (IFN)-induced ubiquitin-specific peptidase 18 (USP18) is a DUB enzyme that negatively regulates the type-I IFN signaling pathway [69]. Two haplotypes of USP18 are linked to MS, and USP18 gene expression is decreased in peripheral blood mononuclear cells and increased during clinical disease activity. USP18 is involved in MS etiology and the IFN-β1b therapeutic response [70]. Table 2 shows the USPs targeted for MS treatment (USP30, USP18, USP16, USP15, etc.). USP18 is also associated with MS pathogenesis.

USP16 is a DUB that has been discovered to be essential for mitotic chromosomal segregation. USP16 aids the correct alignment of chromosomes by promoting the localization and maintenance of polo-like kinase 1 (PLK1) on kinetochores [71]. When combined with the protein regulator of cytokinesis 1, USP16 can deubiquitinate histone H2A and control gene expression (PRC1). USP16 has a novel function and method in regulating mature T cell activation, suggesting that it could be a new therapeutic target for T cell-mediated autoimmune disorders. Zhang et al. looked at USP16 expression in autoimmune illnesses such MS, SLE, and RA to see what function it plays in T cell-mediated inflammation [72].

It is well-known that the NF-κB inhibitor IκBα is degraded via ubiquitin before NF-κB is translocated to the nucleus. USP11 overexpression prevents IκBα ubiquitination. In vitro, recombinant USP11 catalyzes the deubiquitination of IκBα. TNF-induced IB ubiquitination and NF-κB activation are also enhanced when USP11 expression is reduced. These findings show that USP11 modulates IκBα stability and thus plays a crucial role in the downregulation of TNFα-mediated NF-κB activation [73].

USP15 is a member of a large family of USPs that process inactive ubiquitin precursors, remove ubiquitin from cellular adducts and ubiquitinylated proteins, and keep the 26S proteasome free of inhibiting ubiquitin chains. USP15 was found to be capable of deubiquitinating IκBα in order to block TNFα-induced NF-κB activation by the COP9 signalosome [74,75].

Only two DUBs have a transmembrane domain, and USP30 is one of them. Peroxisomes and the outer mitochondrial membrane are the only places where it is found. In cell systems that have been designed to overexpress Parkin, USP30 can restrict Parkin-dependent ubiquitylation of certain substrates and depolarization-induced mitophagy [76]. USP30 prevents TOM20 from being ubiquitylated by Parkin, and its absence increases depolarization-induced cell death in Parkin-overexpressing cells. USP30 also regulates BAX/BAK-dependent apoptosis, and its loss makes cancer cells more susceptible to BH3-mimetics [77].

Through the suppression of NF-kB, the anti-inflammatory enzyme A20, also known as TNF-associated protein 3 (TNFAIP3), is regarded as a crucial gatekeeper in inflammation and peripheral immune system control. The development of various autoimmune and inflammatory illnesses, including MS, has been linked to an A20 malfunction [78].

OTUB1 is a DUB from the OTU family that prefers to cleave Lys48-linked polyubiquitin chains [79]. OTUB1 has a canonical DUB activity that eliminates Lys48-linked polyubiquitin chains directly from substrates, as well as a noncanonical DUB activity that prevents the transfer of Lys48- or Lys63-specific ubiquitin from E2-conjugating enzymes to E3 ligases, preventing target protein ubiquitination. During MS, the DUB OTU domain, ubiquitin-aldehyde-binding 1 (OTUB1), was found to be upregulated in astrocytes [80].

The levels of transcripts encoding E3 ligases such as Cbl-b and ITCH are reduced in the brains of patients with MS, but their levels increase during treatment with IFN, indicating that Cbl-b and ITCH are involved in MS pathogenesis [81,82]. Therefore, molecular targets of the UPS should be further identified; this could provide more insights into the molecular pathogenesis of MS as well as promote the identification of new drug targets.

Both the RAS-MEK-ERK and PI3K-AKT-mammalian target of rapamycin (mTOR) pathways can downregulate or upregulate each other, which suggests a pathway-directed treatment for MS [83]. Thoracic mTOR is a key component sensor [84]. The downstream target of the PI3K/AKT signaling pathway—mTOR—is a catalytic subunit of mTORC1 and mTORC2 complexes that regulates processes such as mRNA translation [85]. The activity of mTORC1 can be regulated by the AKT-activated tuberous sclerosis complex (TSC) in cell membranes. TSC has GTPase-activating protein activity for Rheb (a small GTPase molecule), which directly binds to mTOR and upregulates protein synthesis by activating the kinases 4E-BP1 and p70S6 [86].

### 5.3. UBE2L3 as a Potential Target for Autoimmune Diseases

The E2 ubiquitin-conjugating enzyme UBE2L3 is a potential target for several autoimmune disorders [79,93]. A genome-wide association study has revealed that UBE2L3 is a unique therapeutic target for SLE. The linear ubiquitin chain assembly complex (LUBAC) is required for LUBAC-mediated NF-κB activation, and UBE2L3 is a key E2 enzyme [94]. Overexpressed UBE2L3 increases the activation of NF-κB, which is implicated in the control of inflammatory and autoimmune illnesses [95,96].

## 6. Discussion

When the immune system fails to initiate or maintain tolerance, autoreactive cells become inappropriately activated. Immune tolerance refers to the inability of the immune system to respond appropriately to itself and other harmless antigens, such as allergens. In other words, tolerance mechanisms guarantee that immune cells do not attack the host and are only triggered in response to risks such as injury or exposure to pathogens. Immune tolerance comprises central and peripheral types. Central tolerance develops during lymphocyte maturation [97]. Recent neuropathological and neuroradiological findings have revealed structurally altered axons in plaques and apparently normal white matter in patients with MS. More information about axonal injury in MS is critical, as it might be the main cause of long-term impairment [98].

Because of their propensity to (a) impede nuclear factor (NF)-kB activation and transcriptional modulation of proinflammatory cytokine release and/or (b) prompting apoptosis in triggered immune cells, PIs are another class with potential as investigational drugs. The rationale for using PIs as anti-inflammatory medicines in the treatment of autoimmune illnesses has recently been investigated. The intricate diversity and importance of constitutive and immunoproteasome subtypes in immunologically competent cells in autoimmune disorders as well as numerous classes of reversible and irreversible PIs for therapeutic intervention are discussed herein. Given the chronic nature of MS, the long-term effects of PIs and the possibility of developing resistance to PIs should be investigated [22]. The ubiquitin-independent degradation of MBP by proteasomes appears to occur at physiologically relevant concentrations of MBP [3]. MS is characterized by immune-activated demyelination, neural injury, and plaque development, as well as macrophage, T cell, and B cell invasion. This process is clear in MS, with the accumulation of toxic protein aggregates, indicating UPS malfunction and its probable role in MS pathogenesis. Both protein accumulation and inflammatory responses are aided by the UPS.

Ubiquitination safeguards autoreactive antigens released from the immune system, and if hindered, might trigger autoimmunity. DUBs diminish ubiquitination by either directly eliminating ubiquitin(s) from target proteins or inhibiting the synthesis of ubiquitin chains, which can counteract this dynamic and reversible process. E3 ligases have evolved in critical signaling pathways for the regulation of T cell tolerance toward self-antigens, and their level of ubiquitination is governed by T cell tolerance and apoptosis [99]. While the UPS is required for the degradation of damaged and misfolded proteins, it also participates in lymphocyte growth, activation, and differentiation via the inflammatory process. In addition, the UPS is involved in the regulation of inflammatory factors, such as cytokines and NF-κB. The enzymatic activities of proteasomes are elevated in the CNS of patients with MS. The UPS is a key regulator of the NF-κB activation pathway during the inflammatory response [100].

## 7. Conclusions

Ubiquitination, which is fine-tuned by DUBs, regulates immune responses in CNS autoimmunity. DUBs are becoming popular treatments for various disorders, including cancer and autoimmune diseases. However, given the complex nature of MS, blocking one or more cytokines is usually insufficient, and inhibiting signaling pathways, such as the NF-κB pathway, might cause severe complications because these pathways are essential for preserving normal cellular functions. Inhibitors or agonists of DUBs are sufficiently specific and powerful to reduce neuroinflammation in MS, while avoiding the risk of harmful side effects. More information about DUB expression in immune cells (B cells, T cells, and macrophages), preferably from blood and CSF, along with brain-resident cells in MS lesions, is needed. The development of specialized DUB inhibitors and agonists to treat CNS autoimmune diseases is underway.

## Figures and Tables

**Table 1:** Characteristics of E3 ubiquitin ligase types.

Types	Functional Domain	Members	Reference
HECTs	N and C lobes and flexible lobes in between	NEDD4, ITCH, SMURF1, SMURF2, WWP1, WWP2, UBR5 HERC1, HERC2, HERC3, HERC4, E6AP	[35,36,37]
RINGs	RING folded structure with or without zinc binding domain	c-CBL, E4B, cIAP, CHIP, Mdm2-MdmX, SCF, CRL2s, CRL3s, CRL4s, CRL5s, Cullin7/FBXW8, APC/C	[38,39,40]
RBRs	Two ring domains on terminal with one internal ring domain	HHARI, ARIH2/TRIAD1, NF14/TRIAD2, RNF216/TRIAD3, PARC/ CUL9, ANKIB1, PAPKIN, HOIL-1L, HOIP	[41,42]

HECT, homologous to the E6-AP carboxyl terminus; RING, Really Interesting New Gene; RBRs, RING-between-RING.

**Table 2:** Selected USPs targeted for neuro-autoimmune diseases.

USP	Nature	Characteristics/Signaling	Therapeutic Target	Ref.
USP30	Deubiquitinating enzyme with a transmembrane domain	Mitochondria-anchored DUBs; PINK1/Parkin-mediated mitophagy in cells	Potential target for neuro-autoimmune disease	[87]
USP18	Deubiquitinating enzyme	Acts as a negative regulator of type-I interferon (IFN) signaling; involved in IFN-β signaling	Low level of USP18 Expression is directly related to the severity of MS	[70,88]
USP16	Deubiquitinating enzyme	Deubiquitination of PLK1 and histone H2A to control chromosome function	Specific USP16 inhibitors may be effective in treating MS caused by T cells.	[72]
A20	Deubiquitinating and E3 ligase domains	Encoded through TNFAIP3 gene; crucial gatekeeper of immune homeostasis/ involved in NF-κB signaling	Mutation in TNFAIP3 gene leads to autoimmune diseases including MS	[70,89]
USP15	Deubiquitinating enzyme	Regulates type-I interferon response; activation of the transcription factor NF-κB and regulation of its inhibitor IκBα	Potential target for neurodegenerative diseases	[90]
USP11	Deubiquitinating enzyme	Suppresses TNFα-and stimulates activation of NF-κB by targeting IκBα	DUB inhibitor targets the USP11 and acts as an immunosuppressive drug to protect against multiple sclerosis	[91]
OTUB1	Deubiquitinating enzyme	It inhibits IFN-γ-activated JAK2-STAT1 signaling via Lys48 deubiquitinating and stabilizing SOCS1, the JAK2 inhibitor.	Potent target as T and NK cells are important mediators in MS and OTUB1 hinders the activation of T cells and NK cells	[80,92]

## Data Availability

Not applicable.
